# Prokaryotic Soluble Overexpression and Purification of Human VEGF165 by Fusion to a Maltose Binding Protein Tag

**DOI:** 10.1371/journal.pone.0156296

**Published:** 2016-05-27

**Authors:** Minh Tan Nguyen, Martin Krupa, Bon-Kyung Koo, Jung-A Song, Thu Trang Thi Vu, Bich Hang Do, Anh Ngoc Nguyen, Taewook Seo, Jiwon Yoo, Boram Jeong, Jonghwa Jin, Kyung Jin Lee, Heung-Bum Oh, Han Choe

**Affiliations:** 1 Department of Physiology and Bio-Medical Institute of Technology, University of Ulsan College of Medicine, Asan Medical Center, Seoul 05505, Korea; 2 Osong Medical Innovation Foundation, New Drug Development Center, Division of Drug Screening and Evaluation, Chungbuk, 363–951, Korea; 3 Department of Convergence Medicine, Asan Institute for Life Sciences, University of Ulsan College of Medicine, Asan Medical Center, Seoul, 05505, Korea; 4 Department of Laboratory Medicine, University of Ulsan College of Medicine and Asan Medical Center, Seoul 05505, Korea; Centro Nacional de Biotecnologia - CSIC / CIF Q2818002D, SPAIN

## Abstract

Human vascular endothelial growth factor (VEGF) is a key regulator of angiogenesis and plays a central role in the process of tumor growth and metastatic dissemination. *Escherichia coli* is one of the most common expression systems used for the production of recombinant proteins; however, expression of human VEGF in *E*. *coli* has proven difficult because the *E*. *coli*-expressed VEGF tends to be misfolded and forms inclusion bodies, resulting in poor solubility. In this study, we successfully produced semi-preparative amounts of soluble bioactive human VEGF165 (hVEGF). We created seven N-terminal fusion tag constructs with hexahistidine (His6), thioredoxin (Trx), glutathione S-transferase (GST), maltose-binding protein (MBP), N-utilization substance protein A (NusA), human protein disulfide isomerase (PDI), and the b'a' domain of PDI (PDIb'a'), and tested each construct for soluble overexpression in *E*. *coli*. We found that at 18°C, 92.8% of the MBP-tagged hVEGF to be soluble and that this tag significantly increased the protein's solubility. We successfully purified 0.8 mg of pure hVEGF per 500 mL cell culture. The purified hVEGF is stable after tag cleavage, contains very low levels of endotoxin, and is 97.6% pure. Using an Flk1^+^ mesodermal precursor cell (MPC) differentiation assay, we show that the purified hVEGF is not only bioactive but has similar bioactivity to hVEGF produced in mammalian cells. Previous reports on producing hVEGF in *E*. *coli* have all been based on refolding of the protein from inclusion bodies. To our knowledge, this is the first report on successfully expressing and purifying soluble hVEGF in *E*. *coli*.

## Introduction

Vascular endothelial growth factor (VEGF) plays a critical role in the formation of new blood vessels [1,2]. Deletion of one or two VEGF alleles in mice results in impaired vasculogenesis or death at the mid-gestation stage [3]. Hypoxia is a strong trigger for the production of VEGF and induces angiogenesis [4]. VEGF can be highly expressed in multiple normal physiological states, such as wound healing and the normal female menstrual cycle [5,6]. However, aberrant angiogenesis from overexpression of VEGF results in several diseases such as rheumatoid arthritis, atherosclerotic plaque formation, visual loss in diabetic retinopathy, and tumor angiogenesis [5,7].

Four VEGF homologs, VEGFA, VEGFB, VEGFC, and VEGFD, have been described in the human genome [8]. Several splicing variants of VEGFA, such as VEGF121, VEGF121b, VEGF145, VEGF165, VEGF165b, VEGF189, and VEGF206, have been reported [9]. Among these, VEGF165 is the most abundant and the predominantly active VEGF isoform. It consists of two 165-amino acid monomers, each containing a single glycosylation site and a cysteine knot motif that drives the folding kinetics [9]. The active form of VEGF is a homodimer, and thus, many studies have attempted to obtain a recombinant bioactive homodimer of VEGF.

VEGFs are synthesized and secreted by various cell types including endothelial cells, fibroblasts, macrophages, T cells, neutrophils, platelets, keratinocytes, renal mesangial cells, and many tumor cells [10,11]. Several strategies have been used to heterologously express and produce VEGFs in various eukaryotic expression host systems such as yeast [12,13], insect [14], rice [15], silkworm [16] and mammalian cells [17]. However, the production of VEGF in these systems can lead to impaired hyper-glycosylation and is limited by requiring strict cultivation conditions and specialized equipment.

*E*. *coli* is a convenient platform for protein expression. However, attempts to express VEGF in *E*. *coli* result in low levels of soluble protein expression or protein misfolding and aggregation into inclusion bodies, hampering purification [18,19,20]. Soluble protein expression is favorable compared to refolding protein from inclusion bodies as the overall yield of biologically active protein from inclusion bodies is low [21]. In addition, the need for extensive optimization of the protein refolding conditions makes the refolding process cumbersome. To our knowledge, all biologically active VEGF165 produced in *E*. *coli* to date have been obtained from inclusion bodies.

Protein tags such as thioredoxin (Trx), glutathione S-transferase (GST), maltose-binding protein (MBP), N-utilization substance protein A (NusA), human protein disulfide isomerase (PDI), and the b'a' domain of PDI (PDIb'a'), have been used to enhance the soluble expression of recombinant proteins in *E*. *coli* [22,23,24,25]. Previous studies have reported some success in expressing human VEGF in *E*. *coli* using GST [26], histidine [20] and MBP tags [27]. However, in the case of the former study, the VEGF was refolded from inclusion bodies, while in the latter studies, expression and purification conditions were not optimized. There is a paucity of comprehensive studies that compare the solubility and expression levels of VEGF fused with protein tags. Many proteins lose their solubility or activity after tag cleavage, and the biological activity of VEGF proteins expressed in *E*. *coli* compared to mammalian expression systems after tag cleavage is unknown.

In this study, we created different human VEGF165 (hVEGF) tag fusions and compared their expression levels and solubility in *E*. *coli*. We purify the MBP-hVEGF fusion and show that the MBP tag acts as a general molecular chaperone and promotes proper folding of hVEGF in *E*. *coli*. Finally, we show that the recombinant hVEGF retains its solubility and bioactivity even after tag cleavage, and that the bioactivity is equivalent to that of purified hVEGF from mammalian cells.

## Materials and Methods

### Materials

All the chemicals were analytical grade. Dithiothreitol (DTT) and 1-thio-β-d-galactopyranoside (IPTG) were from Anaspec (Fremont, CA); Coomassie brilliant blue R-250, and Tris-HCl were from Amresco (Solon, Ohio); imidazole was from Daejung Chemicals (Siheung, Korea); ampicillin was from DuchefaBiochemie (Haarlem, Netherlands); NaCl, glycerol, and maltose were from Samchun Chemical (Pyongtaek, Korea); 2-mercaptoethanol was from Yakuri Pure Chemicals (Kyoto, Japan). All the purification columns were purchased from GE healthcare (Piscataway, NJ). PDVF membrane and Acrodisc Syringe Filters, Supor Membrane, were from Pall Corporation (Ann Arbor, MI). Limulus Amebocyte Lysate test kit was from Lonza (Basel, Switzerland). *E*. *coli* Origami 2 (DE3) cell was from Novagen (Madison, WI). Lambda integrase and excisionase were from Elpis Biotech (Daejon, Korea); dialysis membranes were from Viskase (Darien, IL). Anti-hVEGF antibody (Cat. No. sc-507) was from Santa Cruz Biotechnology (Dallas, TX). Peroxidase labeled-anti-rabbit IgG (Cat. No. AbC-5003) was from Abclon (Seoul, Korea). Protein-pak 300SW SEC 7.5 x 300 mm column was from Waters Corporation (Milford, MA). Monoclonal antibodies used in activity assay were from eBioscience (San Diego, CA). ANTI-FLAG M2 affinity gel was from Sigma-Aldrich (St. Louis, MO). Amicon Ultra, leukemia inhibitory factor (LIF), and Accutase were from Merck Millipore (Darmstadt, Germany). Flk-1 MicroBead Kit was from MiltenyiBiotec (BergischGladbach, Germany). Collagen type IV was from BD Biosciences (Franklin Lakes, NJ). Other chemicals were from Sigma (St. Louis, MO).

### Construction of expression vectors

In order to construct hVEGF-expressing vectors, the Gateway cloning technique was used. BP and LR recombination reactions were performed as previously described [23]. Briefly, *E*. *coli-*codon optimized oligonucleotides encoding 165 amino acids of mature hVEGF (NCBI Reference Sequence: BAA78418.1) were synthesized (GenScript, Piscataway, NJ). The tobacco etch virus recognition site (TEVrs), ENLYFQ/G, was also introduced at the N-terminus of hVEGF oligonucleotide. The whole synthesized DNA sequence was then transferred into donor vector pDONOR207 by BP recombination reaction to obtain entry clone named pENTR-hVEGF. Subsequently, LR recombination reactions between the pENTR-hVEGF and the seven pET22b-based destination vectors, pDEST-HGWA (His6), pDEST-HXGWA (Trx), pDEST-HGGWA (GST), pDEST-HMGWA (MBP), pDEST-HNGWA (NusA), pDEST-PDI (PDI) and pDEST-PDIb'a' (PDIb'a') were performed. The fidelity of all the resulting expression clones was verified by sequencing (Macrogen, Daejeon, Korea).

### Bacterial expression culture conditions

*E*. *coli* Origami 2 (DE3) cells (Novagen, Madison, WI) containing seven expression plasmids were inoculated into LB broth and cultured at 37°C with shaking at 200 rpm. A single colony from plated transformants was inoculated into 5 mL of LB medium containing 50 μg/mL ampicillin and 12.5 μg/mL tetracycline and was grown at 37°C overnight. The overnight culture was then transferred ata1:200 ratio into fresh LB medium supplemented with ampicillin and tetracycline and grown in a shaking incubator at 37°C with shaking at 200 rpm. To induce the protein expression, 0.5 mM IPTG was added into culture batch when the OD_600_ reached 0.7 and then, the temperature was maintained at 37°C for 4 h or decreased to 18°C for 18 h. To evaluate the protein expression level, cells were harvested and analyzed by SDS-PAGE using 10% tricine gel.

### Purification and tag removal of recombinant hVEGF from *E*. *coli*

The cultured cells were harvested from 500 mL cultivation by centrifugation at 3,800 × g for 30 min. Cell pellets were resuspended in 50 mL buffer A (20 mM Tris-HCl, pH 8.0, 250 mM NaCl, 5% glycerol (v/v)) and disrupted by ultrasonic cell disruptor JY99-IIDN (Ningbo Scientz Biotechnology, Guangdong, China) until the lysate was completely homogenized. The supernatant from the lysate was collected by centrifugation at 23,000 × g for 30 min and it was used as a crude sample for further purification.

The MBP-hVEGF protein from prepared crude sample was initially purified by MBP chromatography. Filtered supernatant was loaded onto a 2 × 5 mL MBP Trap HP column pre-equilibrated with buffer A. Column was washed with 10–15 column volume (CV) of buffer A to remove excessive non-specifically bound impurities and then protein samples were completely eluted with 5 CV of buffer B (20 mM Tris-HCl, pH 8.0, 5% glycerol (v/v)) containing 50 mM maltose.

Total 20 mL of partially purified MBP-hVEGF protein was treated with TEV protease which was purified as previously described [23] at a ratio of 1:10 (w/w) for proteolytic cleavage. Since the TEV activity might be inhibited by the high salt concentration, protein sample was dialyzed against buffer B to remove remaining salt before the cleavage. The sample was then diluted with buffer B to final volume of 150 mL and reaction was carried out at 18°C for overnight. The cleaved sample was mixed with 50 mL of buffer B containing 4 M NaCl to give the final concentration of 1 M NaCl. After incubation for 1 h, the sample was centrifuged at 23,000 × g for 20 min and 0.2 μm Acrodisc Syringe Filters, Supor Membrane, was used to remove partial aggregation. The clarified sample was immediately applied to 2 × 5 mL HisTrap HP column pre-equilibrated with buffer A. At this step, hVEGF was eluted along with partial MBP tags by washing the column with buffer A containing 100 mM imidazole whereas remaining MBP tags and TEV protease were washed out of the column with buffer A containing 1 M imidazole. The total 30 mL mixture fraction of hVEGF and MBP was desalted by dialyzed against buffer B followed by additional purification with HiTrap Heparin HP column to exclusively remove residual MBP tags. The 5 mL column was pre-equilibrated with buffer B before sample was loaded onto column resulting in efficient separation as followed: MBP tag was removed in flowthrough step but hVEGF was later eluted with buffer B containing 1 M NaCl. The eluted hVEGF was placed into a 3.5 kDa membrane and dialyzed twice with 1X PBS, pH 7.4 (100 fold dilution each time). Finally, hVEGF was lyophilized and kept at -80°C for further experiment. Protein fractions obtained during purification steps were visualized by 15% tricine SDS-PAGE gel. The protein concentrations were measured using the Bradford method with BSA as the standard [28].

### Purification of recombinant hVEGF from mammalian cells

Recombinant hVEGF DNA coding sequence was subcloned into the pFLAG-CMV1 vector and the protein expressed in Chinese hamster ovary (CHO) cells. CHO cell culture media formulation for VEGF-A production consisted of Iscove's Modified Dulbecco's Medium, 5% of fetal bovine serum (FBS), 1% of Antibiotic-Antimycotic 100X, 1 μM of Methotrexate hydrate. To obtain hVEGF, the CHO cell culture media was collected every 5 days for 20 days. It was then centrifuged at 4°C, 8000 rpm for 10 minutes to remove cell debris and filtered through a 0.2 μm membrane.

The filtered sample was purified by column chromatography using ANTI-FLAG M2 affinity gel as previously described [29]. Bound hVEGF proteins were eluted with 1 ml of 0.2 M glycine HCl, pH 2.7, and mixed with 0.1 ml of 1 M Tris-HCl, pH 9.0 to neutralize pH. The CHO-derived hVEGF was finally buffer exchanged into 1X PBS, pH 7.4 and stored at 4°C until used.

### Electrophoresis and quantification of protein expression and solubility level

Protein fractions were prepared as reducing or non-reducing samples by mixing with 5× sample buffer (312.5 mM Tris-HCl, pH 6.8, 50% glycerol, 5% SDS, 0.05% bromophenol blue) with or without 100 mM DTT, respectively, before being loaded onto a tricine SDS-PAGE gel. The protein bands were then visualized by staining with Coomassie brilliant blue R-250.

To figure out the relative expression and solubility levels of fusion proteins, ImageJ image analyzer (http://imagej.nih.gov/ij) was used. The expression level (%) of fusion protein was calculated based on the density ratio of the fusion protein band to the total *E*. *coli* expressed-protein bands. The solubility level (%) was calculated based on the density ratio of the fusion protein band (present in soluble fraction) to the expressed fusion protein (present in total fraction). Background subtraction was done by determining the lowest value of each individual peak on the ImageJ analysis and subtracting it from the highest value. The ImageJ analysis was performed twice on two separate SDS-PAGE gels from two different batches of expression tests.

### Western blot analysis

Protein bands on SDS-PAGE gels were transferred to a PVDF membrane and then the membrane was blocked with blocking buffer (5% skim milk powder, 10 mM Tris, pH 7.6, 150 mM NaCl). To detect the hVEGF protein, the membrane was incubated overnight with rabbit anti-hVEGF antibody sc-507 (1:1000) at 4°C. On the next day, the membrane was rinsed with washing buffer (10 mM Tris, pH 7.6, 150 mM NaCl, 0.1% Tween 20) followed by incubation with peroxidase labeled-anti-rabbit IgG (clone AbC-5003) for more than 2 h at room temperature. The membrane was rinsed with washing buffer and then incubated for 1 min with PicoEPD western blot substrate solution mixture kit according to manufacturer’s instructions (Elpis Biotech, Daejon, Korea).The excess substrate solution was drained and the membrane was covered with clear plastic wrap. The result was visualized by exposing the membrane to X—ray film for 10 to 60 seconds in the dark room.

### Determination of hVEGF purity by HPLC

Size exclusion chromatography-HPLC was performed using a Protein-pak 300SW SEC 7.5 x 300 mm column. The column was equilibrated with 1X PBS buffer, pH 7.4 with more than 10 CVs. The separation was carried out at room temperature with a flow rate of 1 mL/min for 40 minutes. The protein elution peaks were detected at 280 nm and were checked on an SDS-PAGE gel under non-reducing conditions.

### Endotoxin assay

Endotoxin removal of final hVEGF was carried out following previously described protocol [30]. To determine the endotoxin level in purified hVEGF sample, the QCL-1000^™^ Endpoint Chromogenic Limulus Amebocyte Lysate (LAL) Assay kit was performed following the manufacturing instruction. Firstly, 50 μL of hVEGF, standards, and endotoxin-free water were added to each well of a 96 well-plate pre-incubated at 37°C. Then, 50 μL of LAL was also added and mixed with each sample followed by 10 min of incubation at 37°C. Substrate (100 μL) pre-warmed to 37°C was added to each sample and mixed gently. After incubation for 16 min, 100 μL of stop reagent containing 25% v/v glacial acetic acid in water was added to terminate the reaction and the measurement was set at 405–410 nm wavelength by using a spectrophotometer.

### Activity assay

E14tg2a embryonic stem cell (ESC) was a generous gift from Dr. Jun K. Yamashita (Kyoto University). ESCs were maintained in maintenance media (Glasgow's MEM supplemented with 10% KnockOut Serum Replacement, 5% fetal bovine serum (FBS), 1% non-essential amino acids, 1% sodium pyruvate solution, 1% Penicillin-Streptomycin, 0.1 mM 2-mercaptoethanol and 2,000 U/ml LIF. ESCs were cultured on 0.1% gelatin-coated flask at a density of 1,000–2,000 cells/cm^2^ in differentiation medium (α-MEM supplemented with 10% FBS, 1% Penicillin-Streptomycin and 0.1 mM 2-mercaptoethanol) for 96–108 h. Cells were harvested with Accutase and labeled with Flk-1 MicroBead Kit following the supplier’s instructions. Flk1^+^ mesodermal precursor cells (MPCs) were isolated by magnetic-activated cell sorting (AutoMACS Pro Separator, MiltenyiBiotec).

Flk1^+^ MPCs were then subsequently plated on collagen type IV-coated dishes at a density of 5,000cells/cm^2^ and cultured in differentiation medium supplemented with different concentrations of purified *E*. *coli*-derived or CHO-derived hVEGF. After 48 hours, cells were harvested with 0.05% trypsin-EDTA and resuspended at 1 × 10^6^ cells per 100 μL of 0.5% BSA in 1X PBS. Cells were incubated for 30 minutes with the following antibodies: Phycoerythrin-conjugated anti-mouse CD144 (clone eBioBV13), PerCP/eFluor710-conjugated anti-mouse CD31 (clone 390), Allophycocyanin-conjugated anti-mouse CD140b (clone APB5). Analysis was performed by FACSAria II (BD Biosciences). Phase-contrast images were obtained by Axiovert 200M (Zeiss, Jena, Germany).

### Statistics

Experiments were performed with n ≥ 3 and presented as the mean ± standard error (SE). Group means were compared using a Student’s t-test or a one-way analysis of variance (ANOVA). P < 0.05 was considered significant.

## Results and Discussion

### Construction of hVEGF-expressing plasmids and evaluation of protein expression and solubility

**Fig 1:**
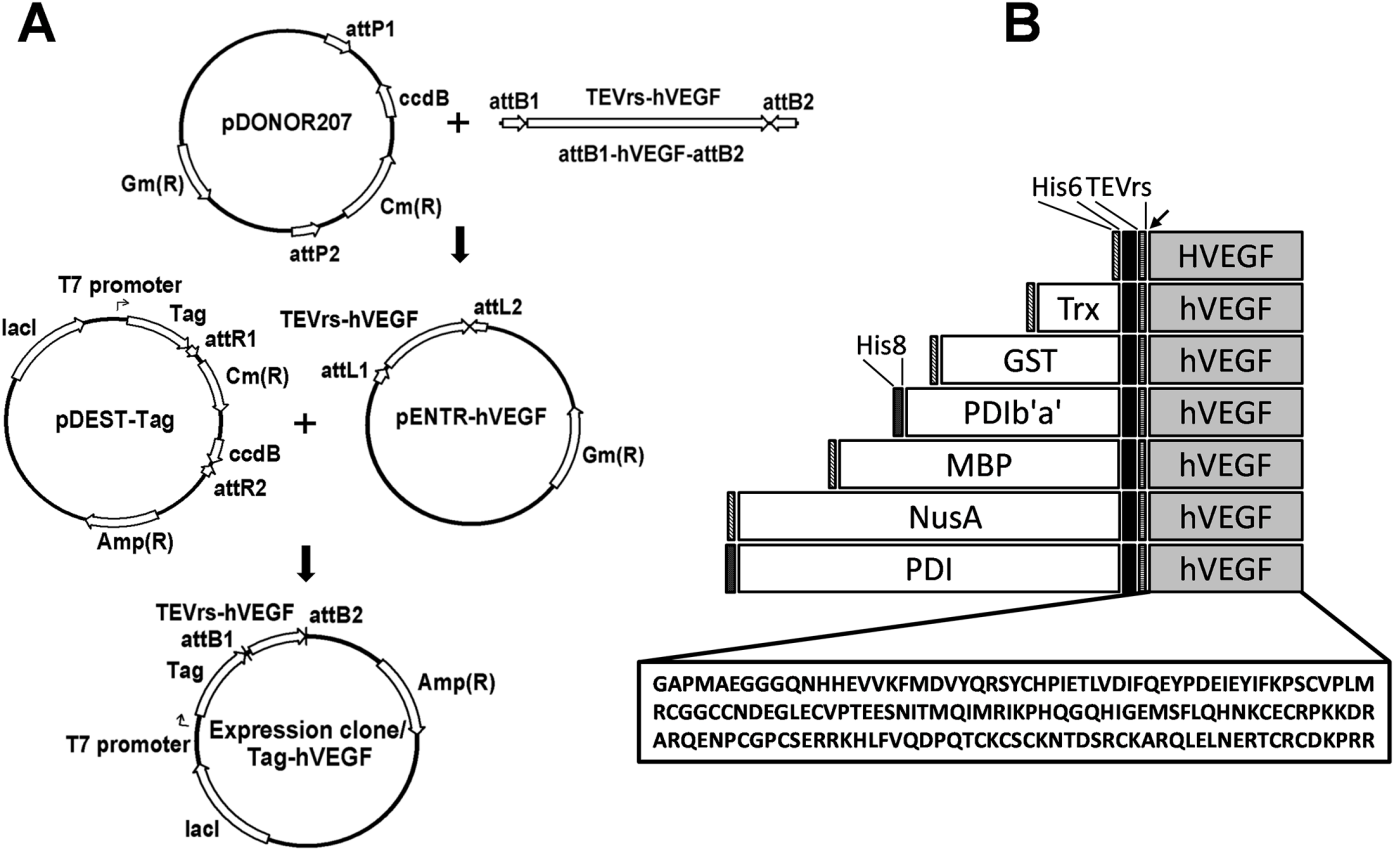
Schematic representation of the domain structure and generation of the MBP-hVEGF construct. (A) Vector map of pHMGWA-hVEGF using the gateway cloning method. BP reaction was done by recombination between attB substrate (attB1-hVEGF-attB2 sequence) and attP substrate (pDONOR207). LR reaction was done by recombination between attL substrate (pENTR-hVEGF) and attR substrate (pDEST-Tag). att, site-specific attachment (att) sites; TEVrs-hVEGF: gene sequence encodes for TEV recognition site and hVEGF protein. Expression of the fusion proteins in *E*. *coli* is driven by the IPTG-inducible T7 promoter with ampicillin as the selection marker.(B) Schematic structure of the seven fusion proteins His6-, Trx-, GST-, MBP-, NusA-, PDIb'a'- and PDI-hVEGF (total size). The arrow indicates the TEV protease cleavage site.

To test the effects of peptide tags on the expression level and solubility of hVEGF in *E*. *coli*, we constructed seven plasmids with N-terminal His6, Trx, GST, MBP, NusA, PDI and PDIb'a' tags. To facilitate subcloning, BP and LR recombinations were used (Fig 1A). A TEV restriction site was inserted between each tag and hVEGF to allow tag separation. The constructs also included His6/His8 tags to facilitate purification (Fig 1B). The sequence of each construct was codon optimized for prokaryotic expression and confirmed, and the constructs were transformed into Origami 2 (DE3) *E*. *coli* cells.

**Fig 2:**
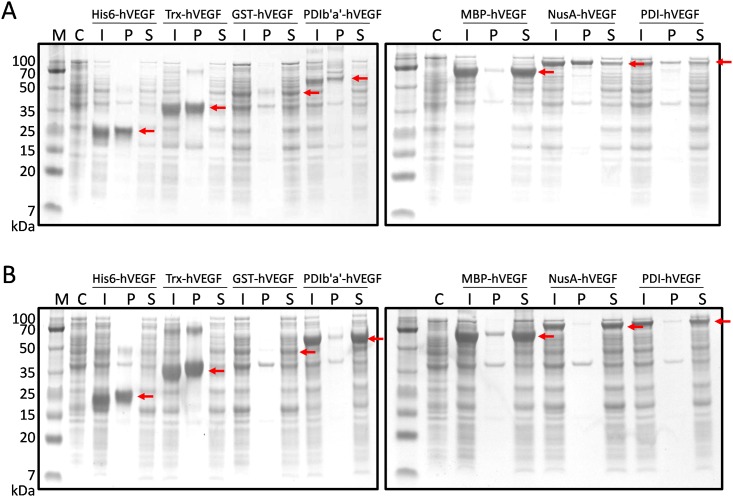
Expression analysis of tagged hVEGF in *E*. *coli* Origami 2 (DE3) by SDS-PAGE. Expression of full-length hVEGF was induced by 0.5 mM IPTG at 37°C (A) and 18°C (B). Arrows indicate the target fusion proteins. His6, hexa (poly) histidine; Trx, thioredoxin; GST, glutathione-S-transferase; PDIb'a', b'a' domain of full-length human PDI; MBP, maltose-binding protein; NusA, N-utilization substance protein A; PDI, full-length human PDI; M, molecular weight marker; C, total cell protein before IPTG induction as negative control; I, total cell protein after IPTG induction; P, pellet fraction after cell sonication; S, soluble fraction after cell sonication.

The hVEGF fusion peptide expression vectors were designed to be driven under the control of the IPTG-inducible T7 promoter. The expression level and solubility varied depending on the tag and temperature. At 37°C, MBP-hVEGF, His6-hVEGF and Trx-hVEGF showed the highest expression levels; each had an expression level greater than 45% (Fig 2A and Table 1). Lowering the expression temperature to 18°C did not significantly increase the expression level of any of the constructs, but did significantly improve the solubility of the NusA-hVEGF, PDI-hVEGF, and PDIb'a'-hVEGF (Fig 2B and Table 1). We chose the MBP-hVEGF construct for further study as this fusion protein showed the highest expression level and solubility at both 37°C and 18°C, and because the MBP tag allowed for straightforward downstream purification. MBP acts as a general molecular chaperone to promote the proper folding of its fusion partner [31]. In addition, MBP is thought to bind to hydrophobic residues present on non-folded proteins, thereby preventing aggregation [32]. MBP is also resistant to proteolysis and may protect its partner protein from degradation [33].

**Table 1:** Expression and solubility level of hVEGF with the seven different tags^a^.

Tag	Tag size (kDa)	Fusion protein size (kDa)	Expression level (%)^b^		Solubility (%)^c^	
			37°C	18°C	37°C	18°C
His6	0.8	22.6	51.0 ± 2.2	49.3 ± 3.4	0.2 ± 0.1	0.2 ± 0.1
Trx	11.7	34.4	45.5 ± 0.1	50.3 ± 4.4	3.0 ± 0.5	3.6 ± 0.4
GST	25.6	48.3	16.7 ± 0.5	16.3 ± 0.4	81.8 ± 1.3	93.5 ± 3.5
PDIb'a'	30.6	53.6	23.8 ± 1.2	42.9 ± 3.8	3.8 ± 0.7	93.3 ± 0.9
MBP	40.2	62.9	53.9 ± 1.4	51.3 ± 0.2	93.0 ± 0.4	92.8 ± 0.1
NusA	54.7	77.5	32.3 ± 0.5	30.4 ± 2.0	39.3 ± 1.9	96.3 ± 0.7
PDI	54.8	77.8	20.9 ± 0.8	28.3 ± 2.5	69.2 ± 6.4	94.7 ± 0.5

^a^ The expression and solubility level were calculated by using densitometry method with two repeats of experiment.

^b^ The expression level (%) of fusion protein was calculated based on the density ratio of target fusion protein to the total *E*. *coli* expressed-proteins.

^c^ The solubility level (%) was calculated based on the density ratio of soluble fusion protein to total fusion protein expressed.

### Purification of hVEGF

**Fig 3:**
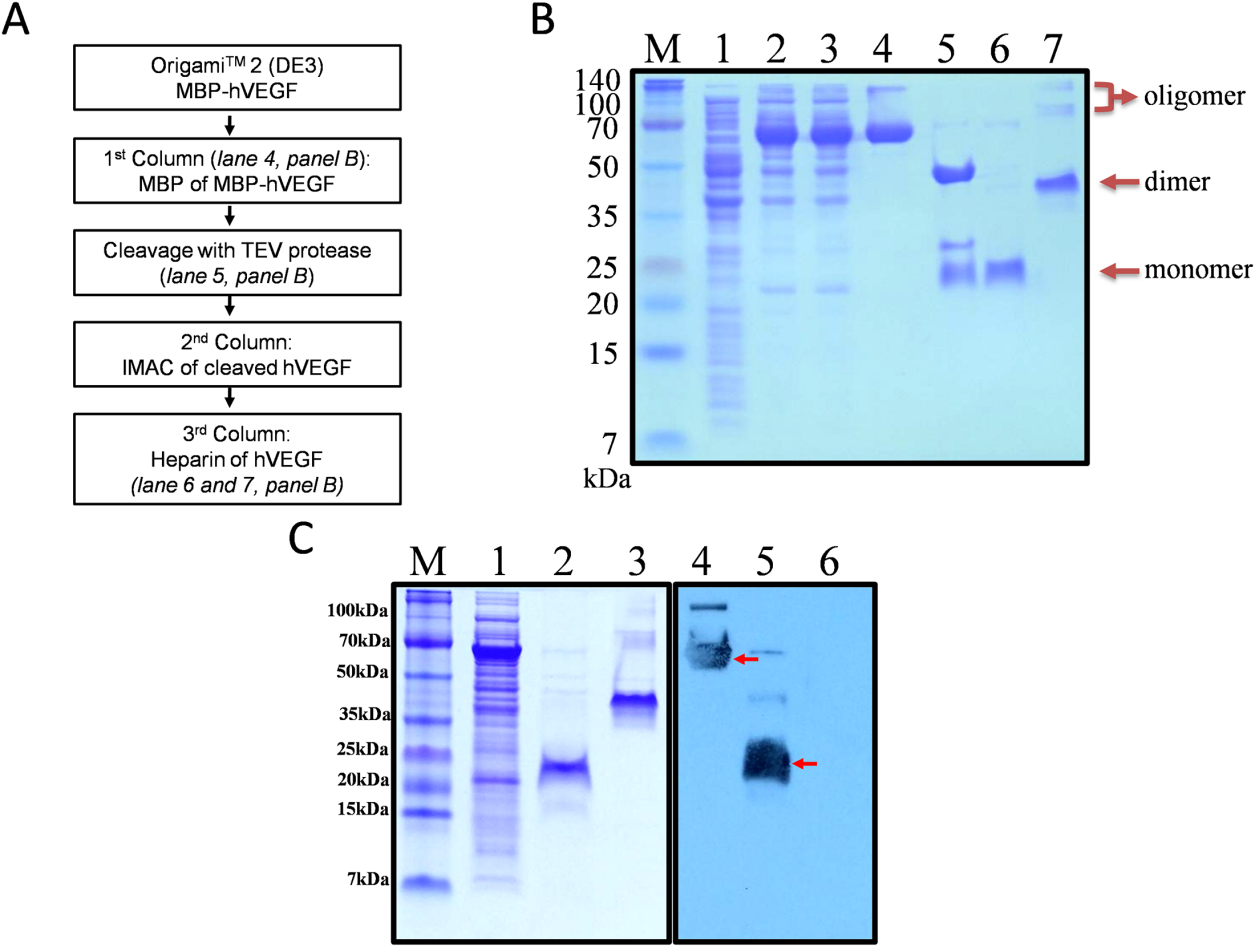
Analysis of hVEGF purification from *E*. *coli*. (A) Schematic overview of hVEGF purification. (B) His6-MBP-hVEGF was purified from *E*. *coli* by chromatography. Lane 5 shows that the MBP tag was almost completely cleaved. M, molecular weight marker; lane 1, total cell extract before IPTG induction as negative control; lane 2, total cell extract with IPTG induction; lane 3, soluble fraction after cell sonication; lane 4, His6-MBP-hVEGF fusion protein purified using MBP column (62.9 kDa); lane 5, His6-MBP tag cleavage with TEV protease (28.6 kDa): His6-MBP (43.9 kDa) and hVEGF (19 kDa); lane 6 and 7, purified hVEGF using Heparin column: final hVEGF product under reducing and non-reducing conditions, respectively. The arrows indicate positions of hVEGF as monomer (19 kDa), dimer (38 kDa) and oligomers (≥100 kDa). (C) SDS-PAGE (lanes 1–3) of reduced and non-reduced samplesand Western blot analysis (lanes 4–6) with anti-hVEGF. The arrows indicate the band signals of hVEGF in fusion with MBP tag or in final product. M, molecular weight marker; lane 1 and 4, total cell extract with IPTG induction; lane 2 and 5, final hVEGF product under reducing condition; lane 3 and 6, final hVEGF product under non-reducing condition.

A 500 mL initial cell culture was lysed and processed in a standard fashion (see Methods) to obtain the MBP-hVEGF containing supernatant, which was loaded onto MBP Trap HP columns. The MBP tag has a high affinity for the dextrin ligand column resins, making it relatively easy to isolate MBP-hVEGF. However, in some cases MBP-fused proteins are not able to bind to the resin due to interactions between the target protein and the MBP tag at the binding site of the tag with dextrin group on the resins [34,35], leading to protein loss in the purification process. As shown in Fig 3B, lane 4, a prominent protein band of ~44 kDa corresponding to MBP-hVEGF was detected in the elution fraction. This suggests that the hVEGF does not occupy the MBP-resin binding site, and thus allows for single-step purification of MBP-hVEGF. TEV protease was used thereafter to cleave the tag off of the partially purified MBP-hVEGF. The tag removal efficiency plays an important step which potentially affects further purification [36]. TEV protease is one of the most well-known viral proteases and is commonly used to cleave protein tags [22,23,24,25]. In this case, the TEV protease was able to completely separate the MBP tag from the hVEGF (Fig 3B, lane 5). Since both the MBP tag and the TEV protease contain a His6 tag in their constructs, a HisTrap HP column was added to the procedure to facilitate the separation of the cleaved tag and the protease from hVEGF. During this step, TEV protease tightly bound to the column and was eluted with high concentration of imidazole; however, partial MBP tag and hVEGF were together eluted earlier.

hVEGF contains a heparin-binding domain and its binding affinity to heparin depends on its properly folded three-dimensional structure [37]. Therefore, a HiTrap Heparin HP column was used to further purify the protein mixture. The MBP tag does not have a heparin-binding domain, and was washed out in the flowthrough step. The SDS-PAGE result indicates that hVEGF sufficiently binds to the column and is efficiently eluted with a buffer containing 1 M NaCl, suggesting that the hVEGF produced in *E*. *coli* is properly folded to expose the heparin-binding domain. Accordingly, the purity of hVEGF estimated by ImageJ image analysis was approximately 97.6%. Fig 3B, lane 6 shows a single band of hVEGF under reducing SDS-PAGE conditions. Non-reducing conditions show that the purified sample contains both, prominent dimer forms and small amount of oligomer forms of hVEGF (Fig 3B, lane 7). SEC-HPLC analysis also showed a major peak corresponding to a dimer form of hVEGF, which was obtained at retention time of approximately 20.5 min (S1 Fig). Approximately 0.8 mg pure hVEGF was obtained from 500 mL of cell culture (Table 2) with endotoxin level less than 0.1 EU/μg. As shown in Fig 3C, lane 4 and 5, under reducing conditions, the hVEGF was detected with an anti-hVEGF antibody. However, there were no signal bands present in a non-reducing condition (Fig 3C, lane 6). This suggests that the folding of hVEGF under non-reducing conditions could hide the binding region of the antibody. For further characterization, a crystal structure analysis could be solved to determine the three-dimensional structure.

**Table 2:** Purification of hVEGF from MBP-hVEGF expressed in *E*. *coli* at 18°C^a^.

Purification step	Volume (mL)	Concentration (mg/mL)^b^	Total protein (mg)^b^	Purity (%)^c^	hVEGF(mg)^d^	Yield(%)
Bacterial culture	500	-	1,950 ± 150 (pellet)	-	-	-
Supernatant	100	1.73 ± 0.06	172.50 ± 5.50	52.2 ± 0.3	27.20 ± 0.70	100 ± 0.0
MBP eluate	20	1.61 ± 0.04	32.20 ± 0.80	96.0 ± 1.0	9.34 ± 0.13	34.4 ± 0.4
IMAC eluate	30	0.69 ± 0.03	20.70 ± 0.90	6.3 ± 1.3	1.31 ± 0.32	4.8 ± 1.0
Heparin eluate	5	0.17 ± 0.01	0.83 ± 0.03	97.6 ± 0.5	0.80 ± 0.02	2.9 ± 0.0

^a^ The results were calculated from 500 mL of cell culture with two repeats of purification.

^b^ Total proteins and protein concentrations were measured by Bradford assay.

^c^ The purity of protein of interest was determined by densitometry using ImageJ.

^d^ The amount of hVEGF was calculated based on the purity of protein of interest and size correlation between hVEGF and fusion protein.

In the purification steps, Tris-Cl buffer was used at 20 mM to resist pH changes due to bacterial components during the lysis step. NaCl at 250 mM was added to buffers to increase ionic strength which has been shown to enhance protein stability and solubility [38]. NaCl and glycerol also prevent weak electrostatic interactions as well as nonspecific hydrophobic protein interactions between the target protein and column with other impurity proteins [39]. A key difficulty during purification reported by many authors is protein aggregation, especially after tag cleavage [40]. For disulfide-rich proteins, the aggregation could be caused by self-association of single protein particles or by newly formed intermolecular disulfide bonds during expression and purification of the target protein [36,41]. We overcame part of this difficulty by keeping sample preparations at a low concentration to eliminate non-covalent interactions of proteins. The cytoplasmic reducing environment of *E*. *coli* cells does not promote the formation of correct disulfide bonds in the target protein. Hence, we used the Origami cell line, which has mutations in thioredoxinreductase (trxB) and glutathione reductase genes [36] that facilitate the formation of correct disulfide bonds. In its active native conformation, hVEGF is a dimer. Our results show that higher order oligomers are also present during purification. These oligomers are prone to aggregate and are likely unstable when on the column, which could harm the heparin resin and affect to the purification. Thus we attempted to remove most of them using a short-term incubation with a high concentration of NaCl followed by Ni-NTA purification. In contrast to the dimer form which was eluted with 100 mM imidazole, the oligomers did not bind to the IMAC column and remained in the flowthrough fraction (data not shown). The overall purification yield after the Heparin column was 2.9% (Table 2). The low final yield comes from the loss of unstable oligomers during purification. Therefore, strategies to increase the amount of synthesized dimer and eliminate unstable oligomers could be beneficial. In addition, the SHuffle strain [42] which has proven to be useful in cytoplasmic synthesis of disulfide bond proteins could be used for expression. Similar to the Origami cell strain, SHuffle cells also contain *trx*B^-^ and *gor*^-^ mutations, but they are engineered to express an additional disulfide bond isomerase DsbC. DsbC is known to act as a chaperone, which is able to break non-native disulfide bonds and feasible to formation of proper ones [43].

### Biological activity of purified hVEGF

**Fig 4:**
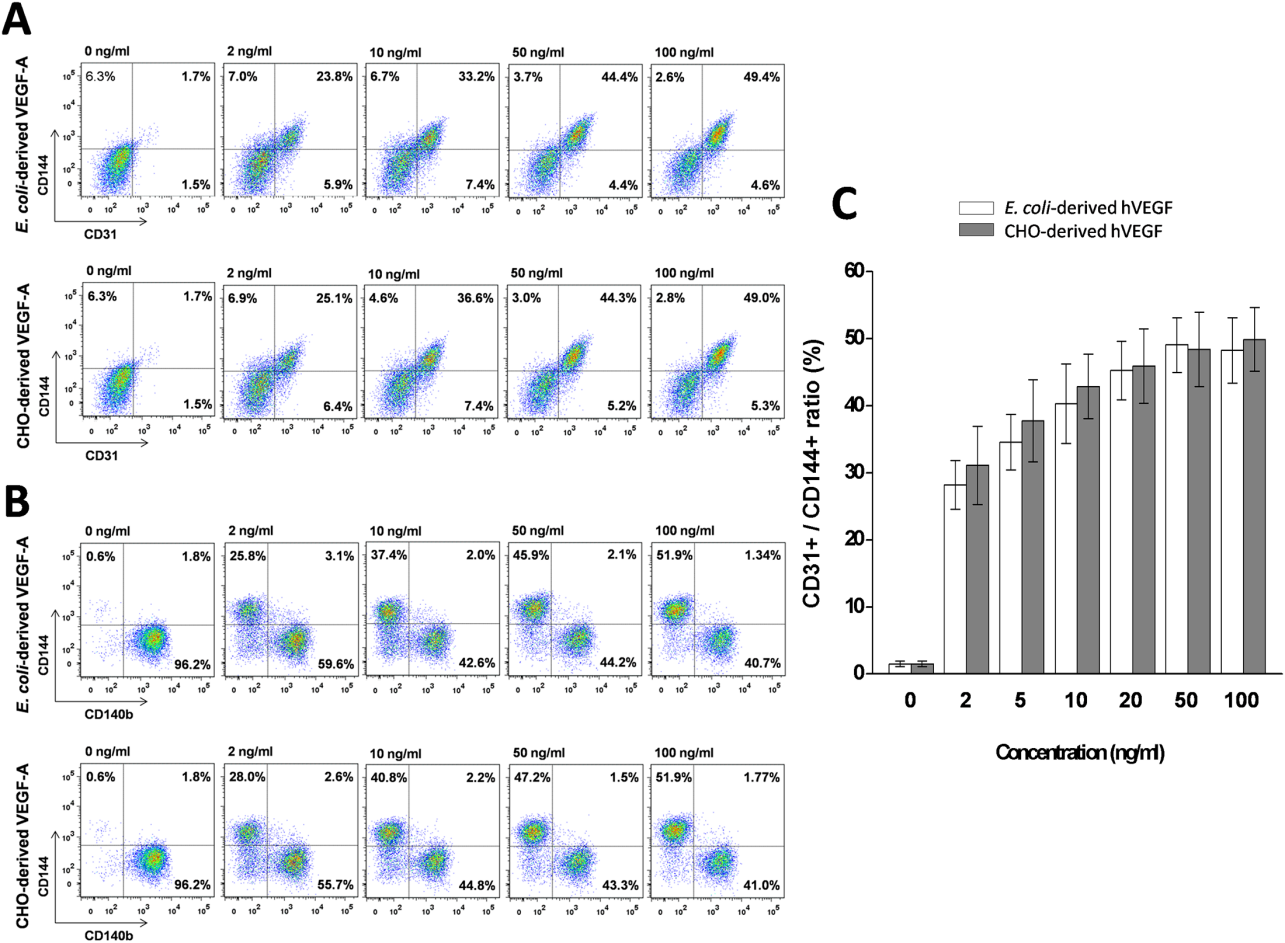
*In vitro* biological activity of purified hVEGF on differentiation of Flk1^+^ MPCs to CD31^+^/CD144^+^ ECs. FACS analysis of Flk1^+^ MPCs incubated with various concentrations of purified hVEGF from *E*. *coli* and mammalian cells for 48 h. (A) The density plots showed the percentage of CD31^+^/CD144^+^ expressing ECs differentiated from Flk1^+^ MPCs. (B) The density plots represented the population of CD144^+^ and CD140b^+^ expressing cells. hVEGF treatment markedly increased the number of CD144^+^ expressing cells, whereas CD140b^+^ expressing cells were reduced. (C) Percentage of CD31^+^/CD144^+^ expressing EC population. Each sample was performed in at least three independent replicates.

The addition of VEGF to a defined medium is known to promote differentiation of Flk1^+^ MPCs to ECs [44]. To confirm the biological activity of the hVEGF derived from our MBP-hVEGF construct, we tested its ability to differentiate Flk1^+^ MPCs into ECs. Flk1^+^ MPCs were incubated with various concentrations of hVEGF obtained from *E*. *coli* as well as CHO cells as a control. The proportion of CD31^+^/CD144^+^ cells increased proportionally to the concentration of hVEGF. More importantly, there was no significant difference between the *E*. *coli* and CHO cell-derived hVEGFs (Fig 4A). Similarly, the proportion of CD140b^+^ and CD144^+^ expressing ECs was affected by hVEGF concentration; CD140b^+^ cells decreased with hVEGF while CD144^+^ cells increased with increasing concentrations of hVEGF (Fig 4B) in a dose dependent manner. However, this proportion was the same for *E*. *coli*-derived and CHO-cell derived hVEGFs, suggesting that *E*. *coli*-derived hVEGF has equal biological activity to hVEGF expressed from mammalian cells in differentiation of Flk1^+^ MPCs into ECs.

Biologically active hVEGF consists of two 165-amino acid monomers, each containing a single glycosylation site and a cysteine knot motif [9]. Initially most hVEGF was produced in mammalian expression systems, such as CHO cells. However, studies have shown that glycosylation does not appear to be critical for bioactivity [45] and that hVEGF expressed in *E*. *coli* retains its biological activity. Unfortunately, the techniques reported for producing hVEGF in *E*. *coli* are based on solubilizing aggregated protein products from inclusion bodies [19,46], which is undesirable due to loss of biological activity and the tedious nature of screening for refolding conditions. Protein yields are not necessarily higher when refolding from inclusion bodies. For example, Lee et al. purified 1 mg hVEGF from an initial 1 L of cell culture with an overall yield of only 1.6% [19]. While this yield is similar to ours (2.9%), the refolding process from inclusion bodies is significantly more time consuming, requiring an additional seven days for protein refolding, as well as complex, requiring the use of complicated refolding buffer components such as stabilizers and redox reagents. The industrial production of hVEGF from inclusion bodies has also been reported with a yield of 30 ± 6% [18]. However, this method requires large industrial refolding instrument, such as a fermenter, as well as, multiple concentration steps after renaturation. In contrast, our method utilizing the MBP fusion tag and standard purification buffer components allows for rapid soluble expression and purification of pure and bioactive hVEGF, without the need for refolding from inclusion bodies.

In conclusion, in this study, we assessed the effect of various tags on the expression level and solubility of hVEGF. We created seven fusion protein constructs with N-terminal His6, Trx, GST, MBP, NusA, PDI, and PDIb’a’ tags and compared their expression and solubility levels at 18°C and 37°C. Both expression level and solubility varied markedly based on tag and temperature (Table 1). Based on the protein expression level, solubility, and size of the fusion protein, we selected the MBP fusion for further purification. Using the MBP fusion construct, we are able to purify 0.8 mg of pure and bioactive hVEGF after tag cleavage from an initial 500 mL of cell culture. Previous reports have focused on either refolding the protein from inclusion bodies or have stopped short at purifying a fusion protein without cleaving the fusion partner. To our knowledge, this is the first report on successfully expressing and purifying hVEGF as soluble form in *E*. *coli*.

## Supporting Information

S1 Fig SEC-HPLC analysis of the final hVEGF. (TIF)

S1 File Supporting Information. (DOC)
