# Correction: Prokaryotic soluble overexpression and purification of human VEGF165 by fusion to a maltose binding protein tag

**DOI:** 10.1371/journal.pone.0170602

**Published:** 2017-01-17

**Authors:** Minh Tan Nguyen, Martin Krupa, Bon-Kyung Koo, Jung-A Song, Thu Trang Thi Vu, Bich Hang Do, Anh Ngoc Nguyen, Taewook Seo, Jiwon Yoo, Boram Jeong, Jonghwa Jin, Kyung Jin Lee, Heung-Bum Oh, Han Choe

In the Funding section, the grant number from the funder Asan Institute for Life Sciences is listed incorrectly. The correct grant number is: 2016–307.
