# Supplementary figures and images for: Prokaryotic Soluble Overexpression and Purification of Human VEGF165 by Fusion to a Maltose Binding Protein Tag
Source: PLoS One. 2016 May 27;11(5):e0156296. doi: 10.1371/journal.pone.0156296 (PMC4883780; doi:10.1371/journal.pone.0156296)

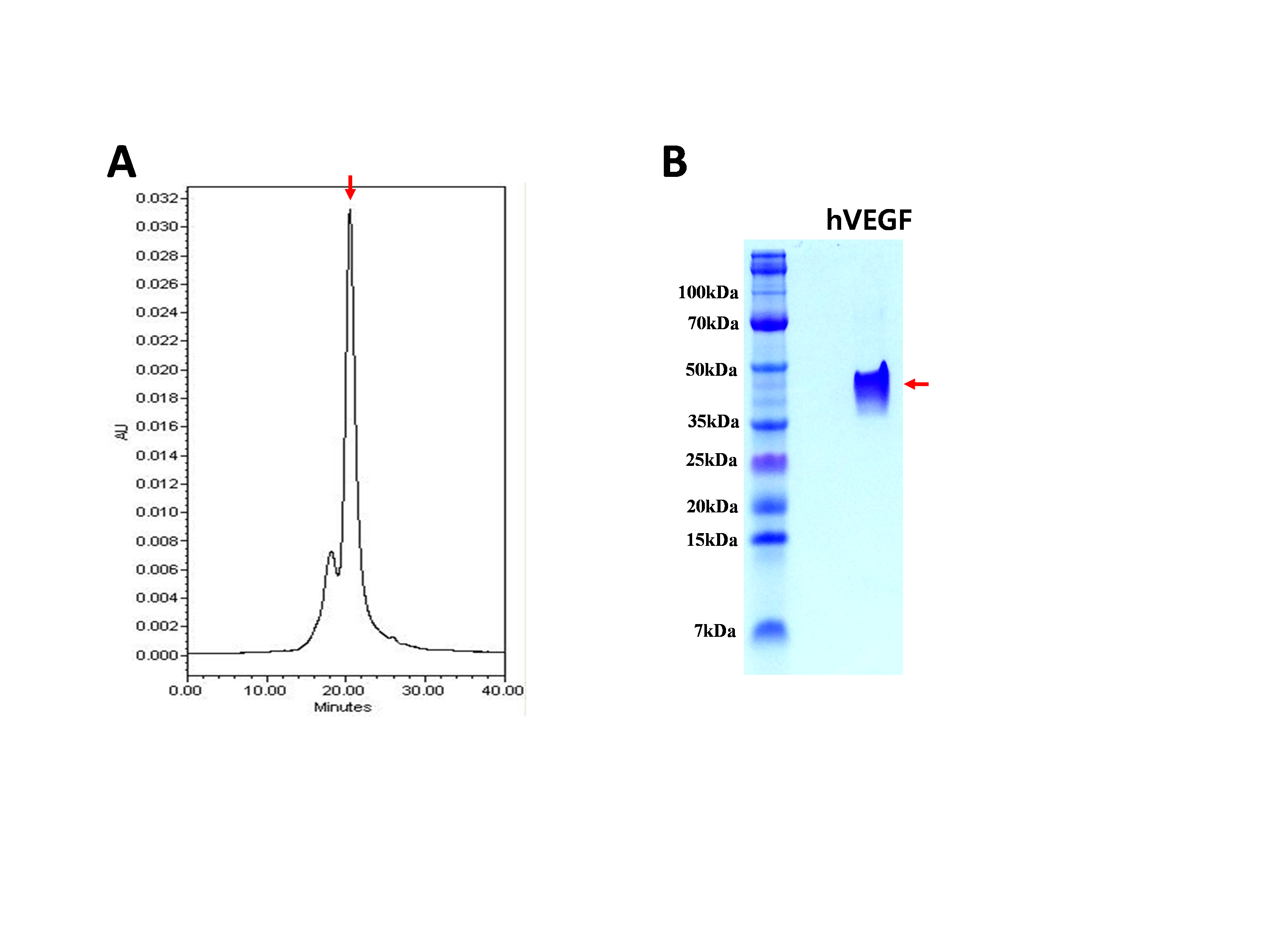

Supplement: S1 Fig — (TIF) [file pone.0156296.s001.tif]
