# Supplementary material for: Prokaryotic Soluble Overexpression and Purification of Human VEGF165 by Fusion to a Maltose Binding Protein Tag
Source: PLoS One. 2016 May 27;11(5):e0156296. doi: 10.1371/journal.pone.0156296 (PMC4883780; doi:10.1371/journal.pone.0156296)
Supplement: S1 File — (DOC) [file pone.0156296.s002.doc]

**Supplementary Figure Legends**

**S1 Fig. SEC-HPLC analysis of the final hVEGF.** (A) Analytical HPLC chromatogram of final hVEGF product. (B) SDS-PAGE of the hVEGF dimer peak in non-reducing condition. The arrow indicates the hVEGF dimer fraction.


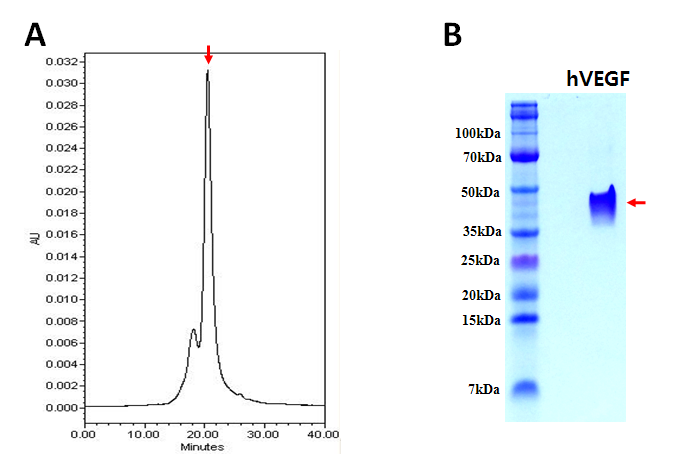


**S1 Fig.**
